# Four New Sesquiterpenoids from the Roots of *Diarthron Tianschanica* with Their Antineoplastic Activity

**DOI:** 10.3390/molecules23061383

**Published:** 2018-06-08

**Authors:** Dong-Xue Sun, Dan Zhao, Hong-Yan Wei, Xiao-Ling Ma, Lei-Ling Shi, Jing Zhang

**Affiliations:** 1 College of Chinese Medicinal Material, Jilin Agricultural University, Changchun 130118, China; sdx51998@163.com (D.-X.S.); zhaodan11954@163.com (D.Z.); 2 Department of Natural Medicinal Chemistry, Xinjiang Institute of Chinese and Ethnic Medicine, Urumqi 830002, China; whywlmq@sina.com (H.-Y.W.); maxiaoling_00@163.com (X.-L.M.)

**Keywords:** thymelaeaceae, *Diarthron tianschanica*, sesquiterpenoids, antineoplastic activity

## Abstract

Four new sesquiterpenoids, known as diarthronchas A–D (**1**–**4**), and one known daphnauranol B (**5**) were isolated from the methanol extract of the roots of *Diarthron tianschanica*. The compounds structures were determined on the basis of spectroscopic data. All of the isolated compounds were profiled for their antineoplastic activity.

## 1. Introduction

**Figure 1:**
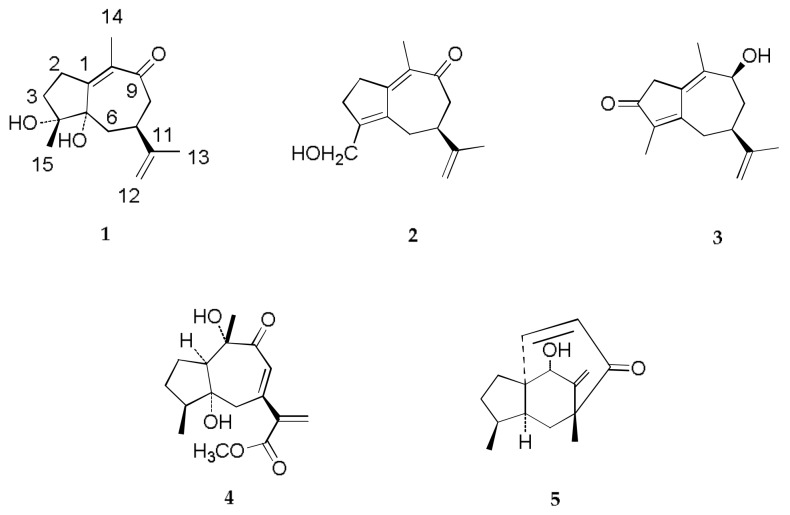
Structures of compounds diarthronchas A–D (**1**–**4**) and daphnauranol B (**5**).

*Diarthron tianschanica (Pobed.) Kit Tanis*, a member of the genus *Diarthron* (Thymelaeaceae), is only observed in the Zhaosu County of Xinjiang Uygur Autonomous Region, China [1]. The roots of *D. tianschanica* have been used in folk medicine to cure a wide variety of ailments, including coughs; asthma [2]; bronchitis [3]; and tuberculosis of the skin, bone, and epididymis [4]. Previously, our group examined the chemical constituents of *D. tianschanica*, resulting in the identification of lignans, coumarins, and diarylpentanols [5,6]. As part of our ongoing phytochemical study, we further investigated the chemical constituents from the roots of *D. tianschanica* and obtained four new sesquiterpenoids, known as diarthronchas A–D (**1**–**4**), and one known daphnauranol B (**5**) [7] (Figure 1). In this paper, we elucidate the structure of these new compounds and their antineoplastic activity.

## 2. Results and Discussion

### 2.1. Purification of Compounds ***1***–***4***

The roots of *D. tianschanica* (10 kg) were soaked with MeOH at room temperature and extracted three times under reflux. The sesquiterpenoids were isolated and purified via silica gel chromatography, Sephadex LH-20 gel chromatography, and semi-preparative high-performance liquid chromatography (HPLC).

### 2.2. Structure Elucidation of Compounds ***1***–***4***

**Figure 2:**
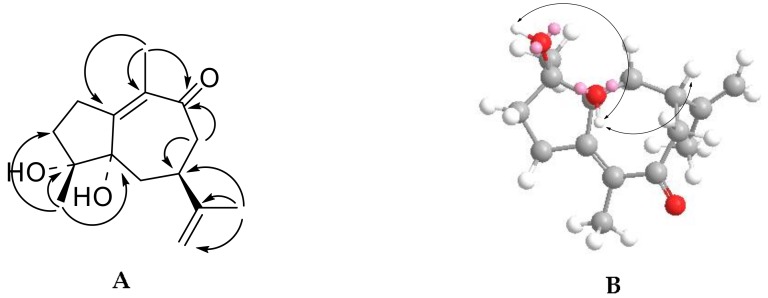
(**A**) Key HMBC (arrows) and (**B**) NOESY (arrows) correlations of compound **1**.

Compound **1** was obtained as a white powder. This compound’s molecular formula was established to be C_15_H_22_O_3_, based on the HRESIMS quasimolecular ion at *m*/*z* 273.1485 [M + Na]^+^ (*Calcd for.* 273.1467 C_15_H_22_O_3_Na). The infrared (IR) and ultraviolet (UV) spectra revealed absorptions for the hydroxyl (3365 cm^−^^1^), and an *α*,*β*-unsaturated ketone moiety (216 nm; 1724 cm^−^^1^) [8]. The ^1^H-NMR spectrum (Table 1) showed three tertiary methyl groups as singlets at *δ*_H_ 1.19 (3H, s, H_3_-15), 1.54 (3H, s, H_3_-14), and 1.72 (3H, s, H_3_-13), and an exo-methylene as two broad doublets at *δ*_H_ 4.65 (1H, d, *J* = 1.8 Hz, H-12a) and 4.71 (1H, d, *J* = 1.8 Hz, H-12b). The ^13^C-APT NMR spectrum revealed 15 carbon signals due to three methyls, five methylenes, one methine, and six quaternary carbons. Among these signals, the carbon signals at *δ*_C_ 136.8 (C-10), 172.1 (C-1), and 205.3 (C-9) indicated the presence of an *α,β*-unsaturated ketone group, and the carbon signals at *δ*_C_ 20.1 (C-13), 108.7 (C-12), and 151.0 (C-11) revealed the existence of an anisopropenyl group [8]. The results described above suggested that compound **1** contained a guaiane-type skeleton [8]. All the signals and functional groups were confirmed by HSQC, HMBC, and ^1^H-^1^H COSY spectra. In the HMBC spectrum (Figure 2A), the protons of *δ*_H_ 1.54 (s, H_3_-14) were correlated with *δ*_C_ 136.8 (C-10), 172.1 (C-1), and 205.3 (C-9), indicating that the connection of H_3_-14 is at C-10 and the presence of an *α*,*β*-unsaturated ketone group. Moreover, the proton signal at *δ*_H_ 1.19 (s, H_3_-15) had long-range correlations with carbon signals at *δ*_C_ 39.5 (C-3), 73.9 (C-4), and 83.3 (C-5), suggesting that the methyl group of H_3_-15 was located at the quaternary carbon C-4. The proton signal at *δ*_H_ 2.67 (1H, m, H-7) was interrelated with *δ*_C_ 151.0 (C-11) and 108.7 (C-12). The double bond protons at *δ*_H_ 4.65 (1H, d, *J* = 1.8 Hz, H-12a) and 4.71 (1H, d, *J* = 1.8 Hz, H-12b) and the methyl protons at *δ*_H_ 1.72 (3H, s, H-13) were connected with *δ*_C_ 42.2 (C-7) and 151.0 (C-11), respectively, suggesting the presence of an isopropenyl group at C-7. Furthermore, the HMBC correlations from *δ*_H_ 4.31 (1H, br s) to *δ*_C_ 73.9 (C-4) and *δ*_H_ 5.27 (1H, br s) to *δ*_C_ 83.3 (C-5) indicated that two OH groups were attached to C-4 and C-5, respectively. The relative stereochemistry of compound **1** was determined by the Nuclear Overhauser Effect Spectroscopy (NOESY) experiment (Figure 2B), in which correlations were observed between the following protons (OH-4/OH-5, H-7/OH-5). Finally, because of the n–π electron transition effect of the *α*,*β*-unsaturated ketone group, the CD spectra of compound **1** showed the cotton effects at 325 nm (Δε −0.7) and 250 nm (Δε + 2.0); thus, the C-5 is in the *S* configuration [9]. Based on these results, compound **1** was elucidated as described and given the trivial name of diarthroncha A.

Compound **2** yielded the molecular formula of C_15_H_20_O_2_, based on the HRESIMS analysis of *m*/*z* 255.1348 [M + Na]^+^ (*Calcd for.* 255.1361 C_15_H_20_O_2_Na). Its ^1^H and ^13^C-NMR (Table 1) data were very similar to **1**, except for the additional double bond and hydroxymethyl groups in **2**. In contrast to **1**, the signal for C-4 and C-5 of compound **2** showed strong downfield chemical shifts to *δ*_C_ 145.5 (C-4) and 144.9 (C-5), indicating a *sp*^2^ double bond between C-4 and C-5 in **2**. Additionally, in the HMBC spectrum, the methylene protons at *δ*_H_ 4.15 (H-15a) and 4.24 (H-15b) had direct correlations with *δ*_C_ 145.5 (C-4), indicating that the methyl group at C-4 in **1** was oxidized to a hydroxymethyl group in **2**. Thus, the structure of **2** was determined as shown and named diarthroncha B.

Compound **3** was obtained as a white powder with the molecular formula of C_15_H_20_O_2_, in agreement with the positive HRESIMS ion peak at *m*/*z* 255.1349 [M + Na]^+^ (*Calcd for.* 255.1361 C_15_H_20_O_2_Na). Comparison of this compound’s ^1^H and ^13^C-NMR data (Table 2) revealed that its structure was similar to that of oleodaphnoic acid [10], except for the additional hydroxyl group at C-9 and methyl group at C-10 in **3**. In the HMBC spectrum, the correlations from *δ*_H_ 4.33 (1H, m, H-9) to *δ*_C_ 41.0 (C-8) and 130.4 (C-10), together with the signal for C-9, revealed a powerful downfield shift to *δ* 70.0 (+ 44.4 ppm), confirming the presence of a hydroxy group at C-9. Additionally, the HMBC correlations of *δ*_H_ 1.87 (H_3_-14) with *δ*_C_ 138.2 (C-1), 70.0 (C-9), and 130.4 (C-10) suggested that the carboxyl group at C-10 in the oleodaphnoic acid was reduced to a methyl group in **3**. In the NOESY spectrum, the cross-peaks of H-7 and H-9 indicated the 9-OH was in *a*,*β*-orientation. Therefore, the structure of **3** was established as shown and named diarthroncha C.

Compound **4** was obtained as a white powder. The compound’s molecular formula of C_16_H_22_O_5_ was determined via HRESIMS analysis of *m*/*z* 317.1348 [M + Na]^+^ (*Calcd for.* 317.1365 C_16_H_22_O_5_Na). The ^1^H-NMR spectrum (Table 2) showed two methyl groups as signals at *δ*_H_ 1.92 (3H, s, H_3_-14) and 0.90 (3H, d, *J* = 7.2 Hz, H_3_-15), an exo-methylene resonance as two broad doublets at *δ*_H_ 6.09 (1H, d, *J* = 1.2 Hz, H-12a) and 6.12 (1H, d, *J* = 1.2 Hz, H-12b), an olefinic proton signal at *δ*_H_ 5.63 (1H, s, H-8), and a methoxy signal at *δ*_H_ 3.62 (3H, s, 13-OMe). Aside from the single methoxy group, the ^13^C-NMR spectrum exhibited 15 carbon resonances, including two methyls, four methylenes, three methines, and six quaternary carbons (one ketone, one carboxymethyl, two olefinic carbons, and two oxygenated carbons).The ^1^H and ^13^C-NMR data of **4** were characteristic of the guaiane-type skeleton with an *α*,*β*-unsaturated ketone (*δ*c 124.5, 154.3, 202.1) and an acrylic ester group (*δ*c 125.6, 146.3, 165.8). In the HMBC spectrum, the correlations of *δ*_H_ 6.09 (1H, d, *J* = 1.2 Hz, H-12a) and 6.12 (1H, d, *J* = 1.2 Hz, H-12b) with *δ*c 146.3 (C-11), 154.3 (C-7), and 165.8 (C-13) suggested that the isopropenyl group at C-7 in the reported guaiane-type skeletons had been oxidized to an acrylic ester group in **4** [8]. Moreover, the proton of *δ*_H_ 5.63 (1H, s, H-8) was interrelated with *δ*c 154.3 (C-7), and 202.1 (C-9) revealed the presence of an *α*,*β*-unsaturated ketone moiety at C-7/8/9. Furthermore, the ^1^H-^13^C long-range signals from *δ*_H_ 3.62(-OCH_3_) to *δ*c 165.8 (C-13) and from *δ*_H_ 1.92 (H_3_-14) to *δ*c 84.3 (C-10) placed the methoxy group at C-13 and the methyl group at C-10. The downfield chemical shifts of C-5 (*δ*c 78.4) and C-10 (*δ*c 84.3), together with the molecular formula above, indicated the presence of OH groups at C-5 and C-10. The CD spectrum of **4** displayed strong cotton effects at 334 nm (Δε −1.2) and 287 nm (Δε + 2.8), which corresponded to the n → π* and π → π* transitions of the unsaturated dienone. On the basis of the CD excitation chirality method for the unsaturated dienone, C-10 possessed the *S* absolute configuration. Taken together with the NOESY spectrum, the structure of **4** was defined as shown and given the trivial name diarthroncha D.

### 2.3. Antineoplastic Activity of Compounds ***1***–***5***

All of the isolated compounds were tested in vitro for their cytotoxic activity against HepG-2, MCF-7, and HeLa human cancer cell lines, with paclitaxel serving as a positive control. The results (Table 3) showed that compounds **1**, **3**, and **5** were moderately cytotoxic against HepG-2 cells with inhibitory concentration 50% (IC_50_) values at 18.9, 22.5, and 20.3 μM, respectively, while compound **2** was weakly cytotoxic with an IC_50_ value of 41.3 μM. Furthermore, compounds **2** and **5** exhibited weak cytotoxicity against HeLa cells with IC_50_ values of 39.6 and 29.6 μM, respectively. None of the compounds had activity against MCF-7 cell lines.

## 3. Materials and Methods

### 3.1. General Experimental Procedures

Optical rotation data were obtained using a Perkin-Elmer 341 digital polarimeter (PerkinElmer, Norwalk, CT, USA). CD spectra were obtained using a JASCO J-815 spectropolarimeter (JASCO, Easton, Md., USA). UV and IR spectra were obtained using Shimadzu UV2550 and FTIR-8400S spectrometers (Shimadzu, Kyoto, Japan), respectively. NMR spectra were obtained using a Bruker AV III 600 NMR spectrometer (Bruker, Billerica, German) with chemical shift values presented as *δ* values and TMS (Tetramethylsilane) as the internal standard. HRESIMS was performed using an LTQ-Orbitrap XL spectrometer (Thermo Fisher Scientific, Boston, MA, USA). Column-chromatography (CC) was performed using silica gel (100–200 mesh, Qingdao Marine Chemical Plant, Qingdao, China) and Sephadex LH-20 (Pharmacia, Uppsala, Sweden). Precoated silica gel GF254 plates (Zhi Fu Huang Wu Pilot Plant of Silica Gel Development, Yantai, China) were used for TLC. All of the solvents used were of analytical grade (Beijing Chemical Plant, Beijing, China).

### 3.2. Plant Material

The roots of *Diarthron tianschanica* were collected in September 2013 from Zhaosu city, Xinjiang Autonomous Region, China, and were identified by Prof Xiao-Guang Jia, Department of Pharmaceutical Chemistry, Xinjiang Institute of Chinese and Ethnic Medicine. A voucher specimen (NO. 13094) was deposited at the Xinjiang Institute of Chinese and Ethnic Medicine.

### 3.3. Isolation and Purification of Compounds ***1***–***5***

The roots of *D. tianschanica* (10 kg) were soaked with MeOH at room temperature (3 × 40 L, 3 h each) and were extracted three times under reflux. Removal of the MeOH under reduced pressure yielded a methanol extract (2189 g). The residue was dissolved in water and extracted with petroleum ether (3 × 1000 mL), chloroform (3 × 1000 mL), ethyl acetate (3 × 1000 mL), and *n*-butanol (3 × 1000 mL), successively. The petroleum ether fraction (108 g) was subjected to CC (column-chromatography) over a silica gel (100–200 mesh, 15 × 60 cm) eluting with a stepwise gradient of CH_2_Cl_2_-MeOH (from 1:0 to 0:1, that is, 100:0, 100:1, 80:1, 50:1, 30:1, 20:1, 15:1, 0:1, *v*/*v*) to yield fractions A–H. Fr. B was prepared using a Sephadex LH-20 column with MeOH to remove pigments, and purified by semi-preparative HPLC of MeOH-H_2_O (60:40, *v*/*v*) as the mobile phase to yield compounds **1** (5.8 mg, *t*_R_ = 18.8 min) and **2** (2.5 mg, *t*_R_ = 31.2 min). Fr. C was purified by semi-preparative HPLC with MeOH-H_2_O (70:30, *v*/*v*) as the mobile phase to yield compounds **3** (3.1 mg, *t*_R_ = 16.7 min), **4** (4.2 mg, *t*_R_ = 28.3 min), and **5** (3.5 mg, *t*_R_ = 37.5 min).

### 3.4. Characterization of Compounds ***1***–***4***

Diarthroncha A (**1**): white powder (MeOH); [α${]}_{D}^{20}$ + 11.6 (c 0.1, MeOH); UV (MeOH) λmax (logε): 216 (3.62) nm, CD (MeOH) 325 nm (Δε −0.7) and 250 nm (Δε + 2.0); IR (film) ν_max_: 3365, 2923, 2845, 1724, 1782 cm^–1^; ^1^H and ^13^C-NMR data (DMSO-*d*_6_), (see Table 1); HR-ESI-MS *m*/*z* 273.1485 [M + Na]^+^.(*Calcd for.* 273.1467 C_15_H_22_O_3_Na).

Diarthroncha B (**2**): white powder (MeOH); [α${]}_{D}^{20}$ + 16.4 (c 0.1, MeOH); UV (MeOH) λmax (logε): 214 (4.83) nm; IR (film) ν_max_: 3372, 2930, 2848, 1721, 1776 cm^–1^; ^1^H and ^13^C-NMR data (DMSO-*d*_6_), (see Table 1); HR-ESI-MS *m*/*z* 255.1348 [M + Na]^+^ (*Calcd for.* 255.1361 C_15_H_20_O_2_Na).

Diarthroncha C (**3**): white powder (MeOH); [α${]}_{D}^{20}$ + 14.2 (c 0.1, MeOH); UV (MeOH) λmax (logε): 212 (3.71) nm; IR (film) ν_max_: 3368, 2932, 2846, 1729, 1786 cm^–1^; ^1^H and ^13^C-NMR data (DMSO-*d*_6_), (see Table 2); HR-ESI-MS *m*/*z* 255.1349 [M + Na]^+^ (*Calcd for.* 255.1361 C_15_H_20_O_2_Na).

Diarthroncha D (**4**): white powder (MeOH); [α${]}_{D}^{20}$ + 17.5 (c 0.1, MeOH); UV (MeOH) λmax (logε): 210 (4.56) nm; CD (DMSO-*d*_6_) 334 nm (Δε –1.2) and 287 nm (Δε + 2.8); IR (film) ν_max_ 3364, 2927, 2839, 1736, 1783, 1233 cm^–1^; ^1^H and ^13^C-NMR data (DMSO-*d*_6_), (see Table 2); HR-ESI-MS *m*/*z* 317.1348 [M + Na]^+^ (*Calcd for.* 317.1365 C_16_H_22_O_5_Na).

### 3.5. Cytotoxicity Assay of Compounds ***1***–***5***

The tested human cancer cell lines were seeded in 96-well plates (10^7^ cells/well), and the compounds were added at various concentrations (2.5, 5, 10, 25, and 50μM, respectively). Keeping the treatment on for 48 h, MTT (0.5 mg/mL) solution was added to each well, which were incubated for a further 4 h at 37 °C. The supernatant was removed, and the formazan crystals were dissolved in DMSO (150 mL) with gentle shaking at room temperature. Finally, the optical density of each well was measured at 570 nm with a microplate reader.

## 4. Conclusions

In conclusion, five sesquiterpenoids were isolated and characterised by spectrometric analysis (1 and 2D-NMR, HRESIMS). Among the isolated compounds, compounds **1**, **3**, and **5** showed moderate cytotoxicity against HepG cells with IC_50_ values at 18.9, 20.3, and 22.5 μM, respectively. Therefore, we believed that this plant was an important source for the diverse structure of sesquiterpenoids and should be further investigated for biological activities.

## Figures and Tables

**Table 1:** ^1^H (600 MHz) and ^13^C-NMR (150 MHz) spectral data for compounds **1** and **2** (in DMSO-*d*_6_).

Position	1		Position	2	
	*δ*c Type	*δ*_H_ (*J* in Hz)		*δ*c Type	*δ*_H_ (*J* in Hz)
1	172.1		1	162.6	
2	27.6	1.50 (1H, dd, 10.8, 9.6) 1.52 (1H, dd, 10.8, 2.4)	2	39.9	2.26 (2H, m)
3	39.5	1.99 (1H, m) 2.48 (1H, m)	3	34.0	2.48 (2H, m)
4	73.9		4	145.5	
5	83.3		5	144.9	
6	34.7	2.53 (1H, m) 2.65 (1H, m)	6	39.6	2.48 (2H, m)
7	42.2	2.67 (1H, m)	7	41.5	3.28 (1H, m)
8	47.9	2.16 (1H, dd, 12.0, 1.8) 2.60 (1H, dd, 12.0, 10.8)	8	49.6	2.81 (2H, m)
9	205.3		9	199.0	
10	136.8		10	134.1	
11	151.0		11	147.3	
12	108.7	4.65 (1H, d, 1.8) 4.71 (1H, d, 1.8)	12	110.8	4.77 (1H, d, 1.8) 4.79 (1H, d, 1.8)
13	20.1	1.72 (3H, s)	13	20.1	1.75 (3H, s)
14	7.9	1.54 (3H, s)	14	2.3	1.80 (3H, s)
15 OH	27.5	1.19 (3H, s) 4.31 (br s) 5.27 (br s)	15 OH	58.8	4.15 (1H, dd, 11.5, 5.4)

**Table 2:** ^1^H (600 MHz) and ^13^C-NMR (150 MHz) spectral data for compounds **3** and **4** (in DMSO-*d_6_*).

Position	3		Position	4	
	*δ*c Type	*δ*_H_ (*J* in Hz)		*δ*c Type	*δ*_H_ (*J* in Hz)
1	138.2		1	47.6	2.33 (1H, m)
2	40.4	2.90 (1H, m) 3.20 (1H, m)	2	29.1	1.09 (1H, m) 1.23 (1H, m)
3	202.8		3	30.9	1.43 (1H, m) 1.85 (1H, m)
4	137.3		4	34.7	2.56 (1H, m)
5	165.5		5	78.4	
6	35.3	2.63 (1H, m) 2.80 (1H, m)	6	35.5	1.76 (2H, m)
7	37.2	2.65 (1H, m)	7	154.3	
8	41.0	1.93 (1H, m) 1.89 (1H, m)	8	124.5	5.63 (1H, d, 1.2)
9	70.0	4.33 (1H, m)	9	202.1	
10	130.4		10	84.3	
11	149.8		11	146.3	
12	110.1	4.71 (1H, s) 4.76 (1H, s)	12	125.6	6.09 (1H, d, 1.2) 6.12 (1H, d, 0.6)
13	20.2	1.75 (1H, s)	13	165.8	
14	17.0	1.87 (1H, s)	14	15.9	1.92 (3H, s)
15	8.2	1.66 (1H, s)	15 MeO	22.2 51.7	0.90 (3H, d, 7.2) 3.62 (3H, s)

**Table 3:** IC_50_ values of compounds **1**–**5** against HepG-2, MCF-7, and HeLa.

Compound	HepG-2	MCF-7	HeLa
	IC_50_ (μM) *		
**Paclitaxel**	1.80 ± 0.26	3.80 ± 0.31	4.25 ± 0.52
**1**	18.9 ± 0.02	48.7 ± 0.39	>50
**2**	41.3 ± 0.13	>50	39.6 ± 0.53
**3**	22.5 ± 0.09	>50	>50
**4**	>50	>50	>50
**5**	20.3 ± 0.24	>50	29.6 ± 0.61

* IC_50_ = inhibitory concentration 50%.

## References

[1] Editorial Committee of Flora of China (1999). Flora of China.

[2] Fan J.W., Yu L., Ma L.Z., Guo N., Gao Q.S., Zhao Q.M., Zhen F.L., Ge F., Wang Q.K., Deng X.M. (2009). Antimycobacterial activity of 29 plants extracts. Chin. Agric. Sci. Bull..

[3] Ai L.Y. (1994). Clinical observation on 297 cases of chronic bronchitis treated by stellera chamaejasme. J. Tradit. Chin. Med..

[4] Zha K.J., Xu G.J., Jin R.L., Xu L.S., Zhang P.Z. (1995). Comparative observation on the inhibitory effect against tuberculous bacillus of Chinese drug Langdu. J. China Pharm. Univ..

[5] Shi L.L., Ma G.X., Gao H.C., Chen Q.C., Yang J.S., Jia X.G., Zhang J. (2016). Diarylpentanol constituents from the aerial part of Stelleropsis tianschanica. J. Asian Nat. Prod. Res..

[6] Shi L.L., Ma G.X., Yang J.S., Gulinar S., Jia X.G. (2016). Chemical constituents from plant of Stelleropsis tianschanica. Chin. Tradit. Herbal Drugs.

[7] Huang S.Z., Li X.N., Ma Q.Y., Dai H.F., Li L.C., Cai X.H., Liu Y.Q., Zhou J., Zhao Y.X. (2014). Daphnauranols A-C, new antifeedant sesquiterpenoids with a 5/6/7 ring system from *Daphne aurantiaca*. Tetrahedron Lett..

[8] Ishihara M., Tsuneya T., Uneyama K. (1991). Guaiane sesquiterpenes from agarwood. Photochemistry.

[9] Xu F.M., Morikawa T., Matsuda H., Ninomiya K., Yoshikawa M. (2004). Structures of new sesquiterpenes and hepatoprotective constituents from the Egyptian herbal medicine *Cyperus longus*. J. Nat. Prod..

[10] Ingert N., Bombarda I., Herbette G., Faure R., Moretti C., Raharivelomanana P. (2013). Oleodaphnoic acid and coriaceol, two new natural products from the stem bark of wikstroemia coriacea. Molecules.

